# Comparative evaluation of moutan pods and moutan barks by HPLC-DAD-ESI-MS/MS technique

**DOI:** 10.1038/s41598-025-04860-1

**Published:** 2025-07-01

**Authors:** Huimin Xiao, Rui Yao, Binbin Liu, Linrui Duan, Jincai Liu, Fen Lin, Siwang Wang, Jinming Gao

**Affiliations:** 1 College of Chemistry and Pharmacy, Northwest A&F University, Yangling, China; 2 Department of Life Science and Medicine, Northwest University, Xi’an, China; 3 Shaanxi Fengdan Zhengyuan Biotechnology Limited Company, Xi’an, China

**Keywords:** *Paeonia suffruticosa* Andrews, Moutan pod, Moutan bark, HPLC-DAD-ESI-MS/MS, Quantitative analysis, Traditional Chinese medicine, Mass spectrometry, Cheminformatics, Natural products, Screening

## Abstract

To evaluate the development and application of a kind of oil peony by chromatographic analysis of the main active components of the waste moutan pods (MP).The methods adopted in this paper, firstly, the quality evaluation method of MP is established by HPLC-DAD-ESI-MS/MS; The second is to compare the types and contents of the main active components of moutan barks (Chinese Pharmacopoeia, MB) with those of the medicinal parts of peony used in oil. HPLC fingerprint method for the extract of moutan pods (EMP) was established. Twenty-one compounds were identified in EMP and their levels determined in EMP and the extract of moutan barks (EMB) by HPLC. The results showed that the components of EMP and EMB were similar, though their contents differed. These findings suggest that EMP may have similar pharmacological effects to EMB. Our findings regarding the similarity and comparison of the chemical components in the EMB and EMP have important practical value for replacing EMB medicine and protecting peony plants from resource depletion caused by root digging.

## Introduction

A fingerprint showing chemical information is widely used for quality control of traditional Chinese medicine (TCM)^1^. The World Health Organization (WHO), U.S. Food and Drug Administration (FDA), and State Food and Drug Administration of China (SFDA) report that fingerprint technology can be used in the process of establishing quality standards for TCM^2^. Fingerprints can be acquired by spectral and chromatographic fingerprinting. Chromatographic fingerprinting methods include high-performance liquid chromatography (HPLC), thin-layer chromatography (TLC), gas chromatography (GC), and capillary electrophoresis (CE). These methods are recognized as routine analysis methods for the identification and qualification of herbal medicines, which are recognized as the routine analysis methods for the identification and qualification of herbal medicines^3^. Currently, the selection of a single or a few specific components as markers for quality assessments is a widely applied strategy^4^. However, according to TCM theory, all of the medicinal compositions have roles in therapeutic effects. Unlike synthetic pharmaceutical drugs, the therapeutic effects of TCM and their preparations exert curative effects based on the synergic effects of their multi-components and multi-targets^5,6^. Thus, obtaining a more comprehensive and global view, which covers most of the active chemical constituents, is valuable for the quality control of traditional medicine^7^.

**Fig. 1:**
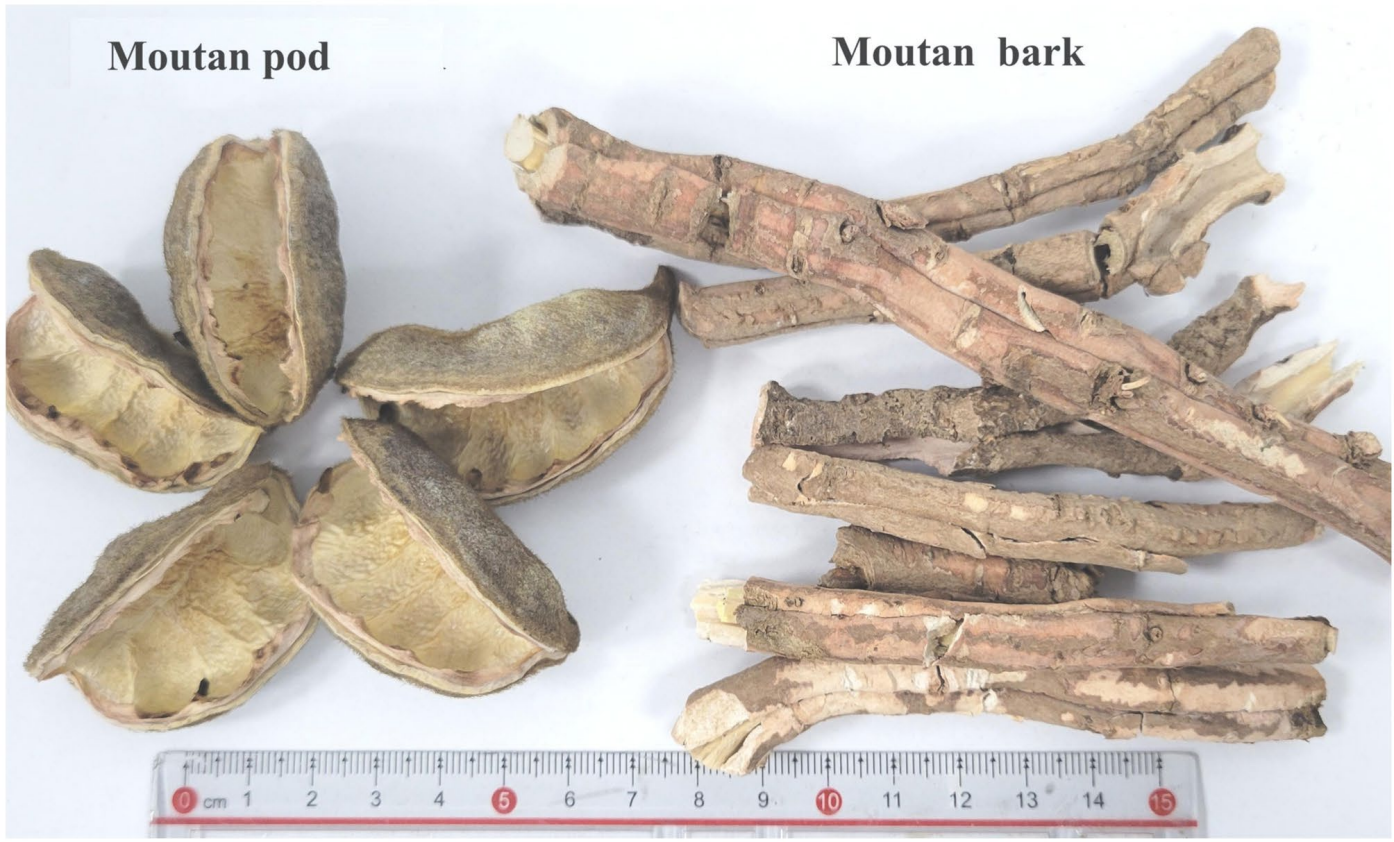
Moutan pod and Moutan bark.

*Paeonia suffruticosa* Andr. is a genus of peony in the goldenseal family. The oil tree peony is an important and extremely rich resource of woody oil worldwide, especially in China. Oil tree peony is a deciduous shrub with ornamental, medicinal and edible values. Its fruit is star-shaped and generally consists of 4–7 pods, which are termed moutan pods (MP, Fig. 1) or seed pods after the seeds have been removed. Moutan bark (MB, Fig. 1) was collected according to the 2020 edition of the Chinese Pharmacopoeia and has a long history of use, with the traditional method for its use involving boiling it in water and taking it orally. MB is a traditional Chinese medicine that functions to clear heat and cool blood, promote blood circulation, and remove blood stasis^8^.

According to the literature^9–19^, the major constituents in MP include the following: 2, 6-dihydroxy-4-methyl benzoate, propyl o-hydroxybenzoate, polysaccharide, cellulose, hemicellulose, lignin, flavonoids, vitamin B6 (VB), gallic acid (GA), catechol (CL), Methyl gallate (MG), hydroxy paeoniflorin (HP), aesculetin (AN), caffeic acid (CA), Paeoniflorin (PF), Proanthocyanidins B2 (PB), paeonin (PN), p-coumaric acid (PA), benzoic acid (BA), ferulic acid (FA), 2,4-dihydroxyacetophenone (DP), gallogen (GL), 1,2,3,6-tetra-O-galloyl-β-D-glucose (GG), 1,2,3,4,6-O-pentagaloyl glucose (PG), apigenin 7-O-neohesperidoside (NP), apigenin 7-O-glucoside (AG), moutonin C (MC), and paeonol (PL). These constituents have been reported to exhibit significant pharmacological activities, including anti-inflammatory, antioxidant, antidepressant, antimicrobial, and anticancer effects, as well as protective effects in the context of malaria, hepatitis, Alzheimer’s disease, Parkinson’s disease, and multiple sclerosis^20–34^. MB has also been reported to contain these major constituents and pharmacological activities^35–46^.

With the vigorous development of the peony industry and its important expansion in the fields of medicine and oil, high volumes of MP waste are generated after its development and utilization; this waste is often disposed of through landfill, incineration, or direct disposal, causing high levels of environmental pollution and representing a great waste of fruit pod resources. Therefore, it is imperative to study the exploitation and utilization of MP.

In conclusion, by establishing an HPLC fingerprint of EMP, we studied the difference in composition and content between EMP and EMB. We hypothesized that EMP and EMB might have similar pharmacological effects, providing a theoretical basis for the full development, utilization, and quality control of EP. Although MPs represent a rich resource, their associated utilization and development research is extremely scarce, resulting in a considerable waste of resources. Our findings relating to the similarity and comparison of the chemical components in the water extracts of MB and MP have important practical value for replacing MB medicine and protecting peony plants from resource depletion caused by root digging.

## Results and discussion

### Optimization of HPLC conditions

**Fig. 2:**
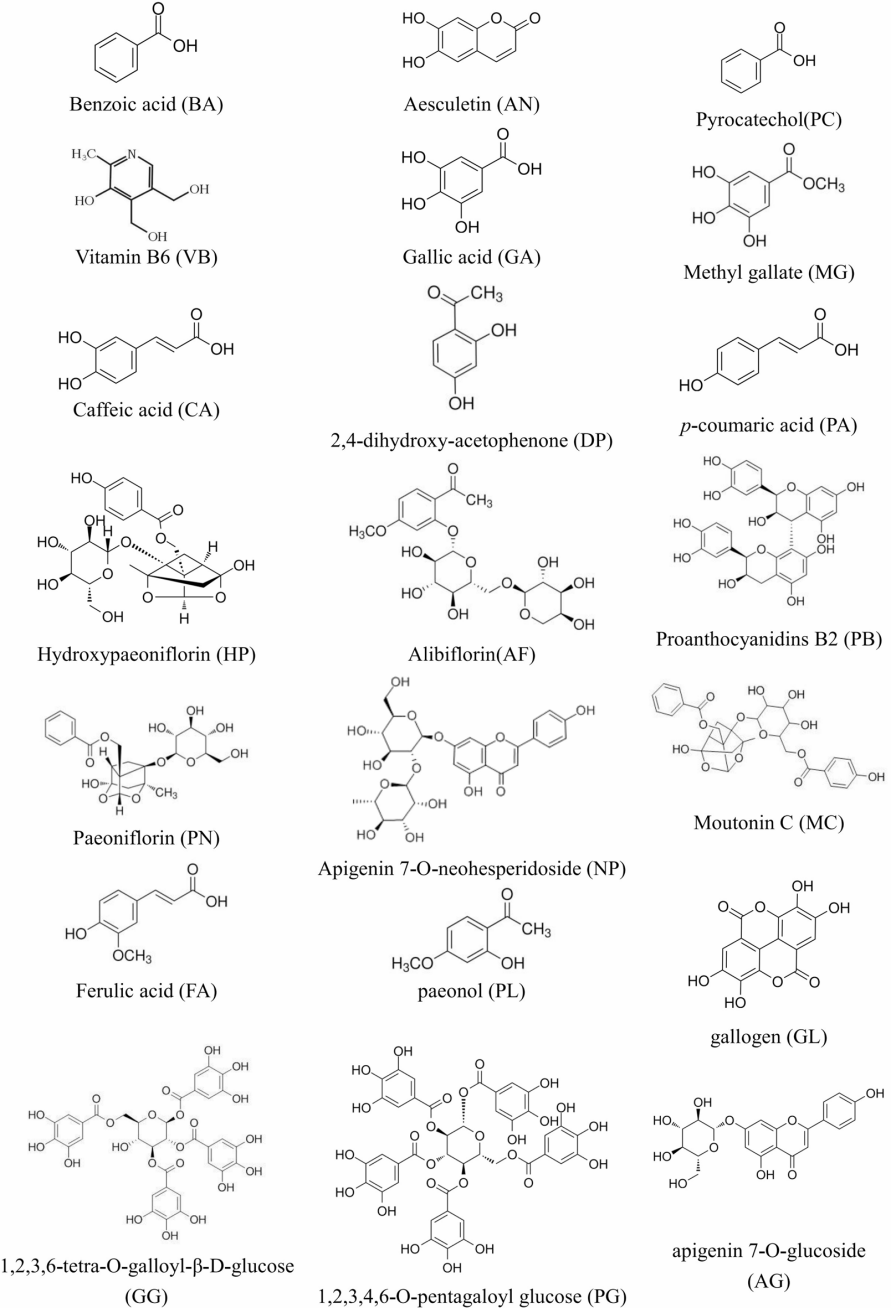
Chemical structures of compounds identified in extracts of moutan pods (EMP) and moutan barks (EMB).

Optimization of the separation conditions for HPLC analysis was performed by identifying a suitable chromatographic column, mobile phase composition, gradient elution program, and detection wavelength. To achieve optimal separation, several trials were attempted, including three kinds of C_18_ reversed-phase columns (Kromasil 100-5-C_18_, Hypersil C18, and Waters C_18_) and different gradient elution systems of acetonitrile–water and methanol–water, with different modifiers, including phosphoric acid, glacial acetic acid, formic acid, and formic acid solutions adjusted by ammonia or triethylamine with different pH values. According to the results, the ShimPack Scepter C_18_ column with a gradient elution system of acetonitrile–0.1% formic acid solution was selected as the preferred chromatographic conditions. The flow rate was 0.8 mL/min and the column temperature was maintained at 25 °C. The DAD was employed at the wavelength range of 190–800 nm to obtain a sufficient number of detectable peaks. The structures of the 21 components are shown in Fig. 2. On the ultraviolet spectra with HPLC chromatograms of the 21 target compounds in EMP, the maximum absorbance values were between 230 and 367 nm. Hence, characteristic chromatographic patterns were obtained by using multi-wavelength as the detection wavelength. The optimal HPLC condition used in this study is shown in section “Method validation of the fingerprints”.

### Method validation of the fingerprints

**Fig. 3:**
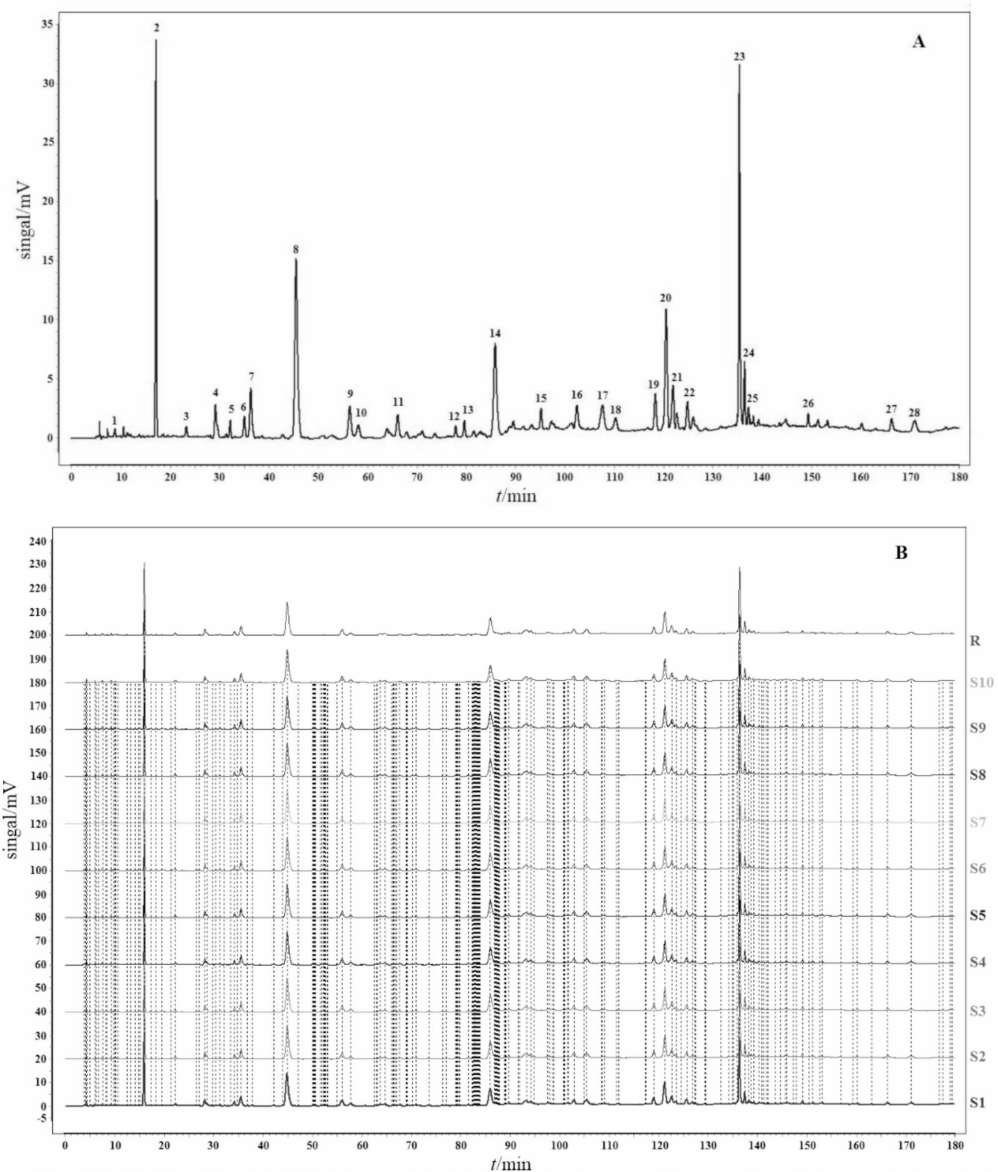
Fingerprint of EMP. (**A**) Representative standard fingerprint. (**B**) Similarities of the fingerprints of 10 samples derived with CASE software.

To obtain a stable and repeatable chromatographic fingerprint of EMP for quality control, the precision, reproducibility, and stability of the method used were expressed by the relative standard deviation (RSD) value of the average relative retention times (RRT) and relative peak areas (RPA) of the 28 common characteristic peaks, using peak 2 as the reference. The precision of the method was obtained by successive analysis of the same sample solution five times. The results demonstrated that the RSDs of precision did not exceed 0.39% for the RRT and 4.39% for the RPA. The reproducibility was evaluated with five independently prepared sample solutions. The results demonstrated that the variation in the RRT and RPA of the charac-teristic peaks did not exceed 0.34% and 4.78%, respectively. The stability test was performed by analyzing the same sample solution at different times (0, 3, 6, 9, 12, 18, and 24 h). The results revealed that the RSDs of stability were below 0.40% for the RRT and 4.96% for the RPA. These results confirmed that the method of HPLC for the fingerprint analysis was both valid and satisfactory (Table 1). To establish the representative HPLC fingerprint of EMP, 10 batches of samples were analyzed, and each chromatogram was used to construct the reference chromatograms. To achieve optimal chromatographic resolution and peak patterns, 28 peaks were screened out as characteristic peaks (Fig. 3A). Similarity is an important parameter for HPLC fingerprinting and comes from the correlation coefficient of the original data.

**Table 1:** Analytical method validation results for the fingerprint analysis.

Peak no	RSD of relative retention time (%)			RSD of relative peak area (%)		
	Precision (n = 5)	Reproducibility (n = 5)	Stability (n = 7)	Precision (n = 5)	Reproducibility (n = 5)	Stability (n = 7)
1	0.26	0.18	0.18	1.43	1.86	2.17
2	0.00	0.00	0.00	0.00	0.00	0.00
3	0.13	0.15	0.15	1.71	4.75	3.23
4	0.08	0.14	0.14	2.52	1.54	2.53
5	0.17	0.06	0.15	1.93	2.94	3.17
6	0.12	0.14	0.14	1.82	1.76	2.18
7	0.35	0.25	0.23	2.18	2.28	4.82
8	0.15	0.16	0.17	2.83	2.95	2.28
9	0.38	0.25	0.33	2.96	3.37	1.97
10	0.17	0.08	0.19	2.76	2.11	2.25
11	0.20	0.20	0.22	2.39	1.56	3.13
12	0.15	0.18	0.18	3.52	1.65	2.65
13	0.15	0.15	0.17	3.17	2.57	3.98
14	0.08	0.22	0.22	2.09	1.84	2.97
15	0.13	0.16	0.19	2.12	1.92	2.75
16	0.18	0.14	0.18	3.05	4.23	3.17
17	0.23	0.23	0.25	3.63	2.46	2.87
18	0.30	0.26	0.39	2.94	1.85	2.56
19	0.22	0.19	0.23	1.97	1.71	1.94
20	0.07	0.26	0.29	4.38	4.75	2.65
21	0.13	0.33	0.14	3.15	1.84	4.95
22	0.14	0.18	0.18	2.15	1.38	1.85
23	0.13	0.20	0.18	3.56	4.19	3.87
24	0.18	0.06	0.09	3.13	3.68	2.77
25	0.22	0.19	0.23	2.75	1.98	3.98
26	0.16	0.17	0.18	1.98	2.39	1.65
27	0.27	0.23	0.08	3.95	4.77	2.79
28	0.16	0.24	0.22	2.76	2.45	2.85

*RSD* relative standard deviation.

The correlation coefficients of 10 batches of EMP were calculated by the Similarity Evaluation System for Chromatographic Fingerprints of TCM (Version 2012) (Fig. 3B). The similarities of the chromatograms of the 10 samples were evaluated by comparing each sample with the reference fingerprint. As shown in Table 2, the quality of sample 2 was lower than that of the other samples. Despite this, the lowest similarity value was only 0.975, which indicated the 10 batches of EMP had a high similarity, as well as no apparent differences in quality.

**Table 2:** Similarities of the chromatograms of 10 batches of EMP.

Similarity	S1	S2	S3	S4	S5	S6	S7	S8	S9	S10	R
S1	1.000										
S2	0.975	1.000									
S3	0.968	0.964	1.000								
S4	0.976	0.973	0.978	1.000							
S5	0.993	0.981	0.967	0.988	1.000						
S6	0.982	0.989	0.981	0.977	0.981	1.000					
S7	0.963	0.992	0.985	0.989	0.985	0.991	1.000				
S8	0.967	0.968	0.975	0.975	0.976	0.985	0.989	1.000			
S9	0.978	0.989	0.996	0.979	0.988	0.977	0.977	0.991	1.000		
S10	0.988	0.991	0.988	0.987	0.991	0.979	0.991	0.989	0.991	1.000	
R	0.976	0.975	0.980	0.983	0.986	0.986	0.987	0.990	0.989	0.987	1.000

### Identification of constituents in EMP

**Fig. 4:**
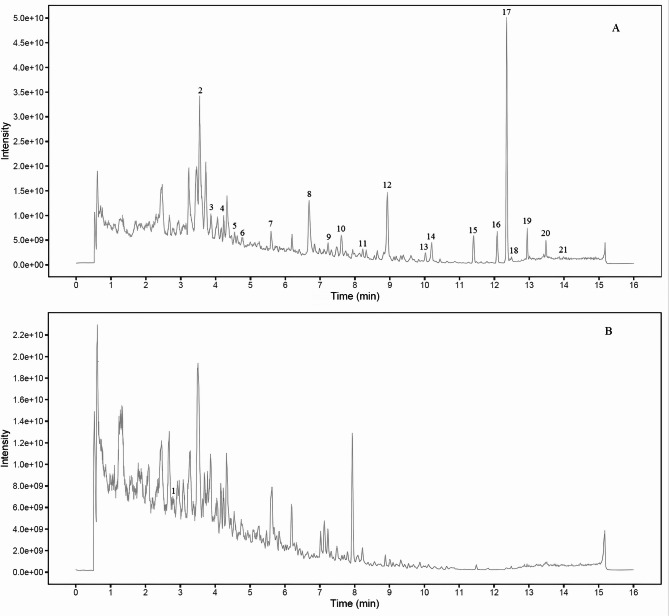
Total ion chromatogram of EMP under different ion modes. (**A**) Negative ion mode. (**B**) Positive ion mode.

**Fig. 5:**
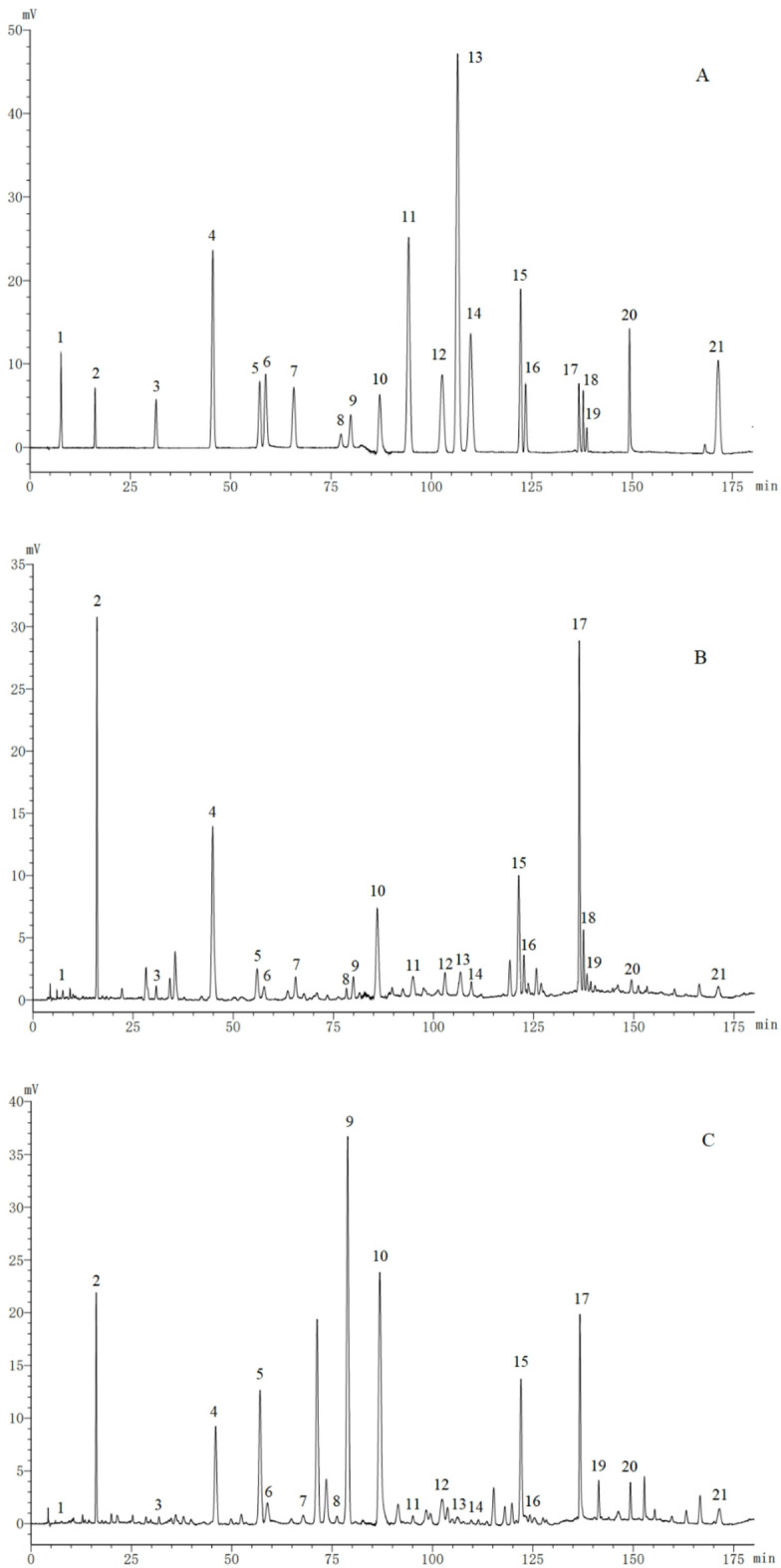
Standard mixture (**A**). EMP sample (**B**). EMB sample (**C**). (1) Vitamin B6; (2) gallic acid; (3) pyrocatechol; (4) methyl gallate; (5) hydroxypaeoniflorin; (6) aesculetin; (7) caffeic acid; (8) alibiflorin; (9) proanthocyanidin B2; (10) paeoniflorin; (11) p-coumaric acid; (12) benzoic acid; (13) ferulic acid; (14) 2,4-dihydroxyacetophenone; (15) gallogen; (16) 1,2,3,6-tetra-O-galloyl-β-D-glucose; (17) 1-2-3-4-6-O-pentagaloyl glucose; (18) apigenin 7-O-neohesperidoside; (19) apigenin-7-O-glucoside; (20) moutonin C; and (21) paeonol.

According to MS/MS data obtained by collision-induced dissociation, 21 components were unambiguously identified by comparing their retention times, MS data, and UV spectra with the reference constituents. Table 3 shows the MS/MS data of the main components. All of the components were detected in positive and negative mode. To further illustrate the characteristic peaks and chemical constitution of EMP, the characteristic chromatograms were compared to those of reference compounds (Fig. 3). According to the fragmentation behavior of the reference standards and consistence in retention times, 21 peaks were unambiguously identified among the 28 characteristic peaks: Vitamin B6 (peak 1), gallic acid (peak 2, reference peak), catechol (peak 5), methyl gallate (peak 8), hydroxypaeoniflorin (peak 9), aesculetin (peak 10), caffeic acid (peak 11), alibiflorin (peak 12), proanthocyanidin B2 (peak 13), paeoniflorin (peak 14), p-coumaric acid (peak 15), benzoic acid (peak16), ferulic acid (peak 17), 2,4-dihydroxyethyl ketone (peak 18), gallogen (peak 20), 1,2,3,6-tetra-O-galloyl-β-D-glucose (peak 21), 1-2-3-4-6-O-pentagaloyl glucose (peak 23), apigenin 7-O-neohesperidoside (peak 24), apigenin-7-O-glucoside (peak 25), moutonin C (peak 26), and paeonol (peak 28) (Table 3 and Figs. 4 and 5).

**Table 3:** Characterization of the components of EMP by LC–MS/MS.

Peak	t_R_ (min)	Formula	Calculated (m/z)	Experimental (m/z)	Error (PSPm)	Ion mode	Main Fragments by LC–MS/MS	Identification
1	2.76	C_8_H_11_NO_3_	169.0214	169.0215	0.59	[M + H]^+^	170.0811, 152.0632, 134.0504 , 94.0401	Vitamin B6
2	3.64	C_7_H_6_O_5_	170.0215	170.0212	–1.76	[M-H]⁻	170.0212, 125.0246, 97.0314, 79.0431	Gallic acid
3	3.92	C_6_H_6_O_2_	110.1113	110.1114	0.91	[M-H]⁻	110.1114, 81.0315, 53.0426, 91.0314	Pyrocatechol
4	4.21	C_8_H_8_O_5_	183.0923	183.0288	–2.73	[M-H]⁻	183.0288, 168.0224, 124.0168, 97.0315	Methyl gallate
5	4.56	C_23_H_28_O_12_	496.1580	496.1583	0.60	[M-H]⁻	496.1583, 333.0411, 165.0212, 121.0346	Hydroxypaeoniflorin
6	4.78	C_9_H_6_O_4_	178.1402	178.1404	1.12	[M-H]⁻	178.1404, 149.0,22, 133.0304, 105.0317	Aesculetin
7	5.66	C_9_H_8_O_4_	180.1572	180.1574	1.11	[M-H]⁻	180.1574,135.0445, 161.0316, 117.0352, 93.0318	Caffeic acid
8	6.81	C_23_H_28_O_11_	480.4623	480.4621	–0.42	[M-H]⁻	480.4621, 317.0512, 121.0331, 165.0205	Albiflorin
9	7.23	C_30_H_26_O_12_	578.5204	578.5212	1.38	[M-H]⁻	578.5212, 289.0305, 245.0436, 125.0242	Procyanidine B2
10	7.82	C_23_H_28_O_11_	480.1631	480.1635	0.83	[M-H]⁻	480.1635, 317.0455, 165.0159, 121.0298	Paeoniflorin
11	8.22	C_9_H_8_O_3_	164.1582	164.1576	–3.66	[M-H]⁻	164.1576, 119.0511, 93.0408, 77.0434	*p*-coumaric acid
12	8.98	C_7_H_6_O_2_	122.1214	122.1216	1.64	[M-H]⁻	122.1216, 77.0423, 93.0334, 105.0316	Benzoic acid
13	10.02	C_10_H_10_O_4_	194.1843	194.1846	1.54	[M-H]⁻	194.1846, 149.0526, 134.0315 , 117.0324	Ferulic acid
14	10.19	C_8_H_8_O_3_	152.1471	152.1467	–2.63	[M-H]⁻	152.1467, 123.0402, 107.0465 , 91.0272	2,4-dihydroxyacetophenone
15	11.45	C_14_H_16_O_8_	302.1932	302.1936	1.32	[M-H]⁻	302.1936, 257.0115,229.1003, 201.0334	Gallogen
16	12.08	C_34_H_28_O_22_	788.5712	788.5726	1.78	[M-H]⁻	788.5726, 635.0885, 483.0411, 313.0222	1,2,3,6-tetra-O-galloyl-β-D-glucose
17	12.19	C_41_H_32_O_26_	940.1182	940.1165	–1.81	[M-H]⁻	940.1165, 769.5591, 599.4381, 169.0142	1,2,3,4,6-O-pentagalloyl glucose
18	12.24	C_27_H_30_O_24_	578.5182	578.5184	0.35	[M-H]⁻	578.5184, 269.0421, 459.0043, 117.0113	Apigenin 7-O-neohesperidoside
19	12.97	C_21_H_20_O_10_	432.1056	432.1055	–0.23	[M-H]⁻	432.1055, 268.0378, 269.0450	Apigenin7-O-glucoside
20	13.51	C_30_H_32_O_13_	600.5688	600.5679	–1.50	[M-H]⁻	600.5679, 437.0843, 479.1024 , 151.0034	Mudanpioside C
21	14.02	C_9_H_10_O_3_	166.1742	166.1744	1.20	[M-H]⁻	166.1744, 137.0324, 150.0315, 93.0314	Paeonol

### Calibration curves and the limit of detection

The mixed standard stock solution containing 21 components was diluted to appropriate concentrations to plot the calibration curves. All of the calibration curves were plotted based on linear regression analysis of the integrated peak areas (y) versus concentrations (x, μg mL^−1^) of the 21 marker constituents in the standard solution at six concentrations. The regression equations, correlation coefficients, and linear ranges for the analysis of the 21 marker constituents are shown in Table 4. All of the analyses showed good linearity (R^2^ > 0.9990) in a relatively wide concentration range. The limit of detection value (LOD) was calculated as the amount of the injected sample that gave a signal-to-noise ratio of 3 (S/N = 3). The LOD values of the method for the 21 marker components are also listed in Table 4.

**Table 4:** Regression equation, linear range, and LODs of the developed method.

Constituent	Regression equation^a^	Correlation coefficient (R^2^)	Linearity range (μg·mL^−1^)	LOD (μg·mL^−1^)
Vitamin B6	*y* = 183.73 *x* – 185.81	R^2^ = 0.9995	3.45–344.96	0.32
Gallic acid	*y* = 1340.37 *x* – 1971.02	R^2^ = 0.9991	3.65–364.56	0.27
Pyrocatechol	*y* = 6834.16 *x* – 1269.37	R^2^ = 0.9992	0.56–55.98	0.04
Methyl gallate	*y* = 55,374.02 *x* + 6725.02	R^2^ = 0.9992	1.12–111.92	0.08
Hydroxypaeoniflorin	*y* = 16,130.90* x* – 1870.93	R^2^ = 0.9996	1.56–155.82	0.14
Aesculetin	*y* = 4221.69 *x* + 5659.19	R^2^ = 0.9995	2.45–245.00	0.22
Caffeic acid	*y* = 18,158.62 *x* + 1517.22	R^2^ = 0.9995	1.08–108.00	0.06
Albiflorin	*y* = 675.89 *x* + 358.62	R^2^ = 0.9996	2.45–245.00	0.21
Procyanidine	*y* = 2404.31 *x* + 871.41	R^2^ = 0.9997	1.86–186.00	0.10
Paeoniflorin	*y* = 15,459.65 *x* + 10,294.19	R^2^ = 0.9996	1.84 ~ 184.04	0.16
*p*-coumaric acid	*y* = 51,941.29 *x* + 3624.17	R^2^ = 0.9997	0.38–38.02	0.05
Benzoic acid	*y* = 4635.62 *x* – 1283.86	R^2^ = 0.9997	1.75–175.03	0.15
Ferulic acid	*y* = 42,894.66 *x* – 2978.08	R^2^ = 0.9998	0.24–24.03	0.02
2,4-dihydroxyacetophenone	*y* = 16,847.79 *x* – 570.85	R^2^ = 0.9996	0.26–25.99	0.03
Gallogen	*y* = 21,934.81 *x* – 4410.73	R^2^ = 0.9997	1.56–156.02	0.14
1,2,3,6-tetra-O-galloyl-β-D-glucose	*y* = 42,822.27 *x* + 2299.73	R^2^ = 0.9993	0.54–54.00	0.04
1,2,3,4,6-O-pentagalloyl glucose	*y* = 10,062.22 *x* – 3552.19	R^2^ = 0.9998	2.35–235.00	0.16
Apigenin 7-O-neohesperidoside	*y* = 17,980.47 *x* + 189.91	R^2^ = 0.9997	0.23–23.01	0.02
Apigenin-7-O-glucoside	*y* = 21,099.92 *x* – 3225.11	R^2^ = 0.9996	0.36–35.99	0.03
Mudanpioside C	*y* = 1370.9755 *x* – 17.9108	R^2^ = 0.9997	0.32–31.99	0.02
Paeonol	*y* = 31,204.75 *x* + 3078.03	R^2^ = 0.9994	0.18–17.99	0.01

^a^y: peak area of components; x: concentration of components.

### Precision, accuracy, stability

The RSD was taken as a measure of precision and accuracy. The precision of the method was evaluated through performing intra- and inter-day assays at three concentrations during a single day and on 5 consecutive days, respectively. As shown in Supplementary Table S1, the overall intra- and inter-day variations were between 0.11%–0.75% and 0.13%–0.87%.

The accuracy tests were conducted using a recovery test by adding three quantities (low, medium, and high) of the 21 standards into samples. The resulting samples were processed as described in section "Calibration curves and the limit of detection" and analyzed using the developed HPLC method. Subsequently, the quantity of each analyte was obtained from the corresponding calibration curve.

The percentage recoveries were calculated according to the following equation: (detection amount—original amount)/added amount × 100%. The results of the calculation revealed that the recovery of all 21 tested bioactive constituents was within the range of 97.23%–101.78%, with a RSD of less than 2%. The results of the precision and recovery test indicated that the method has good precision and accuracy. The proposed HPLC–UV method was successfully applied to simultaneously determine the 21 marker constituents in 10 batches of EMP and 10 batches of EMB. All of the contents are summarized in Supplementary Tables S2 and S3. As shown in Fig. 4 and Supplementary Tables S2 and S3, under these analytical conditions, the 21 marker constituents (VB, GA, PC, MG, HP, AN, CA, PF, PB, PN, PA, BA, FA, DP, EA, GG, PG, NP, AG, MC, and PL) in EMP and EMB could be sufficiently resolved and separated. This method is suitable for routine analysis and contributes to the quality control of EMP and EMB.

### Correlations of EMP and EMB

From Tables S2 and S3, it can be known that the comparison of the number of detected active ingredients: 21 active ingredients were detected in EMP, and 20 active ingredients were detected in EMB (Apigenin 7-O-neohesperidoside was not detected). There was no statistically significant difference between the two. Comparison of the total contents of the detected active ingredients: EMP 129.58 ± 0.83 mg/g, EMB 126.81 ± 0.52 mg/g; There was no statistical difference between the two. It is indicated that the two substances have extremely small differences in the quantity and total content of active ingredients, and they have the same material basis.

## Materials and methods

### Materials

Acetonitrile and methanol (HPLC grade) were purchased from Honeywell (Muskegon, MI, USA); formic acid (HPLC grade, lot number 2020929) was purchased from Tianjin Fuyu Fine Chemical Company Limited; and standards, including VB, GA, pyrocatechol (PC), MG, HP, AN, CA, abiliflorin (AF), PB, paeoniflorin (PN), PA, BA, FA, DP, GL, GG, PG, NP, AG, MC, and PL, were purchased from the National Institute for the Control of Pharmaceutical and Biological Products (Beijing, China). The purity of these compounds was determined to be more than 98% by normalizing the peak areas detected by HPLC, and was shown to be highly stable in methanol solution. Deionized water was prepared from a Millipore water purification system (Milford, MA, USA) and filtered with a 0.22 μm membrane. All of the other reagents were of analytical grade. The MP and MB (MP and MB are from the Shaanxi Heyang Peony Plantation of Shaanxi Heyang Zhongzi Guoye Biotechnology Limited Company CN. Their collection time is on August 10, 2023) were provided by Shaanxi Fengdan Zhengyuan Biotechnology Company Limited.

### HPLC instrumentation and chromatographic conditions

All of the analyses were performed on an Shimadzu 2030C Plus HPLC system and Nexera XR HPLC system (Shimadzu Corporation, Japan), equipped with a quaternary pump, an online degasser, and a column temperature controller, coupled with a DAD (Shimadzu Corporation, Japan) as the detector. Mettler Toledo Electronic Analytical Balance (Model XPE105, METTler); Ultrasonic generator (Model KQ-5200D numerical control, Kunshan Ultrasonic Instrument Co., Ltd.). All of the separations were conducted on a ShimPack Scepter -C_18_ reserved-phase column (250 mm × 4.6 mm, 5 μm, Shimadzu Corporation, Japan). The mobile phase included (A): acetonitrile and (B): 0.1% formic acid aqueous solution with gradient elution (0–20 min, 3%–7% A; 20–60 min, 7% –10% A; 60–105 min, 10%–16% A; 105–125 min, 40%–19% A; 125–130 min, 19%–22% A; 130–151 min, 22%–29% A; 151–170 min, 29%–31% A; and 170–180 min, 31%–57% A), with a 10-min re-equilibration duration between individual runs. The flow rate of the mobile phase was 0.8 mL•min^−1^ and the injection volume was 5 μL. The column temperature was maintained at 25 °C. The components were quantified based on peak areas at the maximum wavelength in their UV spectrum.

### UHPLC-MS/MS instrumentation and chromatographic conditions

LC–MS/MS analysis was performed on an UHPLC system (Vanquish, Thermo Fisher Scientific) with a Waters UPLC BEH C_18_ column (1.7 μm, 2.1 × 100 mm). The flow rate was set at 0.5 mL/min and the sample injection volume was 5 μL.

The mobile phase consisted of 0.1% formic acid in water (A) and 0.1% formic acid in acetonitrile (B). The multi-step linear elution gradient program was as follows: 0–11 min, 85%–25% A; 11–12 min, 25%–2% A; 12–14 min, 2%–2% A; 14–14.1 min, 2%–85% A; and 14.1–16 min, 85%–85% A.

An Orbitrap Exploris 120 mass spectrometer coupled with Xcalibur software was employed to obtain the MS and MS/MS data based on the IDA acquisition mode. During each acquisition cycle, the mass range was from 100 to 1500; the top four of every cycle were screened and the corresponding MS/MS data were further acquired. The parameters were as follows: sheath gas flow rate, 35 Arb; Aux gas flow rate, 15 Arb; ion transfer tube temp, 350 °C; vaporizer temp, 350 °C; full ms resolution, 60,000; MS/MS resolution, 15,000; collision energy, 16/32/48 in NCE mode; and spray voltage, 5.5 kV (positive) or −4 kV (negative).

### Preparation of standard solutions

A standard stock solution containing the 21 components (VB, GA, PC, MG, HP, AN, CA, AF, PB, PN, PA, BA, FA, DP, GL, GG, PG NP, AG, MC, and PL) was prepared in 70% methanol aqueous solution. Working standard solutions, containing the 21 compounds were prepared by appropriate dilution of the stock solution. All of the stock and working standard solutions were stored in brown bottles at 4 °C before analysis.

### Preparation of EMP and EMB samples

To prepare the samples, 5 kg of MP (or MB) was extracted and crushed, before adding 10 × water to reflux extract for 1 h. Following extraction, the sample was filtered and the filtrate was collected. The filtrate residue was extracted by reflux again by adding 10 × water for 1 h. Next, the sample was filtered, combined with the secondary filtrate, concentrated to a clear paste with a relative density of 1.05–1.15 (60 °C), and then spray-dried to obtain a dry powder of EMP or EMB.

### Preparation of EMP and EMB sample solutions

The EMP (or EMB, 0.4 g) was weighed precisely and dissolved in 100 mL of 70% methanol aqueous solution. Subsequently, the solution was extracted with ultrasonication (power: 300 W, frequency: 50 kHz) for 45 min and then settled to a volume of 100 mL. The solution was centrifuged at 12,000×*g* for 15 min, before filtering with a 0.22-μm microporous membrane prior to analysis. Finally, 5 μL of sample solution was aliquoted and injected into the HPLC system for analysis.

## Conclusion

In the present research, chromatographic fingerprint analysis and simultaneous quantitative determination of 21 marker components in EMP were performed using HPLC–UV, and 20 components in EMB were also determined by HPLC–UV. Twenty-eight characteristic fingerprint peaks were selected to evaluate the similarities between 10 batches of EMP. HPLC-DAD-ESI-MS/MS was conducted to determine the structures of the characteristic common peaks. Comparing their mass spectra and retention behavior to those of reference standards or literature data, we identified and quantitatively determined the structures of 21 characteristic constituents.

The results clearly demonstrated that the proposed method was reasonable in terms of linearity, repeatability, precision, stability, and recovery, and thus is fit for the routine analysis of EMP and EMB. The HPLC fingerprint analysis and the precise quantity of the marker components in EMP could provide valuable quantitative information for the quality assessment of EMP. Furthermore, the detection of the major constituents lays the groundwork for performing an in-depth study to further screen the active components of EMP and EMB. Although the components of EMP and EMB are similar, their contents differ. These findings suggest that EMP may have similar pharmacological effects compared to EMB. EMB is a legally prescribed medicinal material included in the pharmacopoeia(Chinese Pharmacopoeia Volume I, 2025 Edition), and its components and medicinal effects have been well studied. EMP contain the indicator components of EMB, such as paeonol, and other major active components, such as paeoniflorin and gallic acid, etc.^20–34^. These components have extensive pharmacological effects and form the material basis of the pharmacological effects of EMB^35–46^.Therefore, the development and application of MPs have promising prospects. Whether moutan pods can be used instead of moutan barks remains to be determined, and we plan to continue to study the predictions based on their material basis and pharmacological aspects.

## Supplementary Information

Supplementary Information.

## Data Availability

The datasets used and/or analyzed during the current study are available from the corresponding author on reasonable request.
