# Supplementary material for: Comparative evaluation of moutan pods and moutan barks by HPLC-DAD-ESI-MS/MS technique
Source: Sci Rep. 2025 Jul 1;15:21739. doi: 10.1038/s41598-025-04860-1 (PMC12218210; doi:10.1038/s41598-025-04860-1)
Supplement: Supplementary file 1 — Supplementary Information. [file 41598_2025_4860_MOESM1_ESM.doc]

**Supplementary information**

**Supplementary Table S1. Precision and recovery data of the proposed HPLC method.**

| Components | Nominal Concentration (μg·mL^−1^) |  | Precision | | | |  | Recovery | |
| --- | --- | --- | --- | --- | --- | --- | --- | --- | --- |
|  |  |  | Intra-day (n = 5) | | Inter-day (n = 5) | |  | Mean ± SD (%) | RSD/% |
|  |  |  | Mean ± SD (μg·mL^−1^) | RSD (%) | Mean ± SD (μg·mL^−1^) | RSD/% |  |  |  |
| Vitamin B6 | 3.45 |  | 3.42 ± 0.02 | 0.58 | 3.43 ± 0.02 | 0.59 |  | 98.58 ± 1.26 | 1.28 |
|  | 68.99 |  | 68.87 ± 0.11 | 0.31 | 67.32 ± 0.12 | 0.18 |  | 100.39 ± 0.93 | 0.93 |
|  | 275.97 |  | 274.15 ± 0.98 | 0.36 | 273.75 ± 1.03 | 0.38 |  | 98.97 ± 0.87 | 0.88 |
| Gallic acid | 3.65 |  | 3.64 ± 0.02 | 0.55 | 3.62 ± 0.01 | 0.28 |  | 98.65 ± 1.19 | 1.21 |
|  | 72.91 |  | 72.23 ± 0.35 | 0.48 | 72.15 ± 0.34 | 0.47 |  | 100.11 ± 0.95 | 0.95 |
|  | 291.65 |  | 290.02 ± 1.12 | 0.39 | 289.71 ± 1.23 | 0.43 |  | 99.65 ± 0.76 | 0.77 |
| Pyrocatechol | 0.56 |  | 0.55 ± 0.001 | 0.19 | 0.54 ± 0.002 | 0.37 |  | 99.56 ± 1.45 | 1.46 |
|  | 11.20 |  | 11.03 ± 0.04 | 0.37 | 10.89 ± 0.03 | 0.28 |  | 101.02 ± 1.07 | 1.06 |
|  | 44.78 |  | 44.07 ± 0.08 | 0.19 | 43.99 ± 0.06 | 0.14 |  | 99.78 ± 0.99 | 1.00 |
| Methyl gallate | 1.12 |  | 1.13 ± 0.006 | 0.53 | 1.11 ± 0.004 | 0.36 |  | 100.12 ± 1.68 | 1.68 |
|  | 22.38 |  | 22.24 ± 0.08 | 0.36 | 22.19 ± 0.09 | 0.41 |  | 98.78 ± 0.82 | 0.83 |
|  | 89.54 |  | 88.95 ± 0.13 | 0.15 | 88.89 ± 0.11 | 0.13 |  | 99.54 ± 0.75 | 0.76 |
| Hydroxypaeoniflorin | 1.56 |  | 1.53 ± 0.002 | 0.13 | 1.51 ± 0.003 | 0.20 |  | 99.89 ± 1.58 | 1.59 |
|  | 31.16 |  | 31.01 ± 0.09 | 0.29 | 30.33 ± 0.12 | 0.40 |  | 101.16 ± 0.93 | 0.92 |
|  | 124.66 |  | 123.73 ± 0.66 | 0.54 | 123.54 ± 0.71 | 0.58 |  | 99.66 ± 0.88 | 0.89 |
| Aesculetin | 2.45 |  | 2.43 ± 0.01 | 0.41 | 2.41 ± 0.01 | 0.42 |  | 101.45 ± 1.44 | 1.42 |
|  | 49.00 |  | 49.03 ± 0.09 | 0.19 | 48.87 ± 0.13 | 0.27 |  | 99.49 ± 0.81 | 0.82 |
|  | 196.00 |  | 195.79 ± 0.25 | 0.11 | 195.16 ± 0.57 | 0.30 |  | 97.35 ± 0.73 | 0.75 |
| Caffeic acid | 1.08 |  | 1.07 ± 0.003 | 0.28 | 1.06 ± 0.004 | 0.38 |  | 100.08 ± 1.98 | 1.98 |
|  | 21.60 |  | 21.37 ± 0.08 | 0.38 | 21.25 ± 0.07 | 0.33 |  | 99.36 ± 0.72 | 0.73 |
|  | 86.40 |  | 85.74 ± 0.19 | 0.23 | 85.35 ± 0.23 | 0.27 |  | 98.54 ± 0.84 | 0.86 |
| Albiflorin | 2.45 |  | 2.46 ± 0.01 | 0.41 | 2.44 ± 0.011 | 0.45 |  | 101.45 ± 1.31 | 1.30 |
|  | 49.00 |  | 48.89 ± 0.09 | 0.19 | 48.85 ± 0.12 | 0.25 |  | 99.49 ± 0.89 | 0.90 |
|  | 196.00 |  | 195.12 ± 1.05 | 0.54 | 195.03 ± 1.10 | 0.57 |  | 98.65 ± 0.65 | 0.66 |
| Procyanidine | 1.86 |  | 1.84 ± 0.007 | 0.38 | 1.82 ± 0.009 | 0.49 |  | 99.86 ± 1.71 | 1.72 |
|  | 37.20 |  | 36.92 ± 0.17 | 0.46 | 36.84 ± 0.21 | 0.58 |  | 97.82 ± 0.77 | 0.79 |
|  | 148.80 |  | 147.95 ± 0.46 | 0.31 | 147.87 ± 0.59 | 0.40 |  | 101.78 ± 0.86 | 0.85 |
| Paeoniflorin | 1.84 |  | 1.83 ± 0.008 | 0.44 | 1.81 ± 0.009 | 0.50 |  | 99.74 ± 1.43 | 1.44 |
|  | 36.81 |  | 36.55 ± 0.21 | 0.58 | 36.28 ± 0.24 | 0.66 |  | 98.81 ± 0.98 | 1.00 |
|  | 147.23 |  | 146.84 ± 0.29 | 0.20 | 146.57 ± 0.33 | 0.23 |  | 97.23 ± 0.62 | 0.64 |
| *p*-coumaric acid | 0.38 |  | 0.37 ± 0.002 | 0.54 | 0.32 ± 0.002 | 0.56 |  | 98.38 ± 1.78 | 1.81 |
|  | 7.60 |  | 7.56 ± 0.02 | 0.27 | 7.52 ± 0.04 | 0.54 |  | 100.76 ± 0.75 | 0.75 |
|  | 30.42 |  | 29.97 ± 0.14 | 0.47 | 29.92 ± 0.16 | 0.54 |  | 98.42 ± 0.84 | 0.86 |
| Benzoic acid | 1.75 |  | 1.73 ± 0.01 | 0.58 | 1.71 ± 0.011 | 0.64 |  | 99.75 ± 1.56 | 1.57 |
|  | 35.01 |  | 34.97 ± 0.12 | 0.35 | 34.89 ± 0.15 | 0.43 |  | 101.01 ± 0.64 | 0.64 |
|  | 140.02 |  | 139.86 ± 0.74 | 0.53 | 139.65 ± 0.81 | 0.58 |  | 97.82 ± 0.87 | 0.89 |
| Ferulic acid | 0.24 |  | 0.23 ± 0.001 | 0.44 | 0.23 ± 0.002 | 0.87 |  | 101.68 ± 1.07 | 1.06 |
|  | 4.81 |  | 4.75 ± 0.02 | 0.43 | 4.72 ± 0.03 | 0.64 |  | 97.81 ± 0.58 | 0.60 |
|  | 19.22 |  | 18.98 ± 0.12 | 0.64 | 18.87 ± 0.13 | 0.69 |  | 100.22 ± 0.69 | 0.69 |
| 2,4-dihydroxyacetophenone | 0.26 |  | 0.26 ± 0.001 | 0.39 | 0.25 ± 0.002 | 0.80 |  | 99.26 ± 1.32 | 1.33 |
|  | 5.20 |  | 5.11 ± 0.02 | 0.40 | 5.02 ±0.03 | 0.60 |  | 100.62 ± 0.91 | 0.91 |
|  | 20.79 |  | 20.63 ± 0.11 | 0.54 | 20.52 ± 0.13 | 0.64 |  | 97.79 ± 0.67 | 0.69 |
| Gallogen | 1.56 |  | 1.55 ± 0.008 | 0.52 | 1.54 ± 0.009 | 0.59 |  | 98.56 ± 1.19 | 1.21 |
|  | 31.20 |  | 31.03 ± 0.21 | 0.68 | 30.95 ± 0.22 | 0.71 |  | 99.32 ± 0.95 | 0.96 |
|  | 124.82 |  | 123.91 ± 0.56 | 0.46 | 123.75 ± 0.75 | 0.61 |  | 100.82 ± 0.84 | 0.84 |
| 1,2,3,6-tetra-O-galloyl-β-D-glucose | 0.54 |  | 0.53 ± 0.003 | 0.57 | 0.53 ± 0.04 | 0.76 |  | 99.54 ± 1.81 | 1.82 |
|  | 10.80 |  | 10.76 ± 0.08 | 0.75 | 10.67 ± 0.07 | 0.66 |  | 98.38 ± 0.74 | 0.76 |
|  | 43.20 |  | 42.94 ± 0.14 | 0.33 | 42.86 ± 0.16 | 0.38 |  | 100.82 ± 0.83 | 0.83 |
| 1,2,3,4,6-O-pentagalloyl glucose | 2.35 |  | 2.34 ± 0.01 | 0.43 | 2.33 ± 0.006 | 0.26 |  | 99.35 ± 1.45 | 1.46 |
|  | 47.00 |  | 46.89 ± 0.15 | 0.32 | 46.75 ± 0.16 | 0.35 |  | 98.47 ± 0.93 | 0.95 |
|  | 188.00 |  | 187.34 ± 1.25 | 0.67 | 187.17 ± 1.36 | 0.73 |  | 101.88 ± 0.71 | 0.70 |
| Apigenin 7-O-neohesperidoside | 0.23 |  | 0.22 ± 0.001 | 0.46 | 0.23 ± 0.001 | 0.44 |  | 99.23 ± 1.64 | 1.66 |
|  | 4.60 |  | 4.56 ± 0.011 | 0.25 | 4.43 ± 0.013 | 0.29 |  | 98.26 ± 0.77 | 0.79 |
|  | 18.41 |  | 18.38 ± 0.12 | 0.66 | 18.31 ± 0.13 | 0.71 |  | 100.41 ± 0.86 | 0.86 |
| Apigenin-7-O-glucoside | 0.36 |  | 0.35 ± 0.002 | 0.57 | 0.35 ± 0.003 | 0.86 |  | 99.36 ± 1.24 | 1.25 |
|  | 7.20 |  | 7.14 ± 0.02 | 0.28 | 7.13 ± 0.03 | 0.42 |  | 97.92 ± 0.79 | 0.81 |
|  | 28.79 |  | 28.15 ± 0.12 | 0.43 | 28.09 ± 0.14 | 0.50 |  | 101.79 ± 0.74 | 0.73 |
| Mudanpioside C | 0.32 |  | 0.31 ± 0.002 | 0.65 | 0.32 ± 0.002 | 0.63 |  | 99.32 ± 1.28 | 1.29 |
|  | 6.40 |  | 6.23 ± 0.03 | 0.48 | 6.31 ± 0.04 | 0.64 |  | 100.64 ± 0.72 | 0.72 |
|  | 25.59 |  | 24.97 ± 0.15 | 0.60 | 25.02 ± 0.13 | 0.56 |  | 97.59 ± 0.61 | 0.63 |
| Paeonol | 0.18 |  | 0.17 ± 0.001 | 0.59 | 0.18 ± 0.001 | 0.56 |  | 99.18 ± 1.17 | 1.18 |
|  | 3.60 |  | 3.54 ± 0.01 | 0.29 | 3.56 ± 0.01 | 0.28 |  | 97.63 ± 0.95 | 0.98 |
|  | 14.39 |  | 14.12 ± 0.06 | 0.43 | 14.13 ± 0.07 | 0.50 |  | 100.39 ± 0.67 | 0.67 |

**Supplementary Table S2. Contents of the 21 components of EMP across 10 batches (n = 3, mean ± SD, mg.g^−1^).**

| Component | S1 | S2 | S3 | S4 | S5 | S6 | S7 | S8 | S9 | S10 | Mean |
| --- | --- | --- | --- | --- | --- | --- | --- | --- | --- | --- | --- |
| Vitamin B6 | 2.4589 ± 0.0079 | 2.4568 ± 0.0125 | 2.4591 ± 0.0102 | 2.4640 ± 0.0316 | 2.4602 ± 0.0118 | 2.4812 ± 0.0098 | 2.4671 ± 0.0192 | 2.4589 ± 0.0086 | 2.4590 ± 0.0154 | 2.4590 ± 0.0067 | 2.4624 ± 0.0073 |
| Gallic acid | 67.3194 ± 0.2817 | 67.1276 ± 0.3572 | 67.3418 ± 0.2186 | 67.3183 ± 0.3105 | 67.2277 ± 0.2418 | 67.3007 ± 0.1187 | 67.1792 ± 0.2943 | 67.1583 ± 0.3342 | 67.3219 ± 0.3562 | 67.3347 ± 0.2852 | 67.2630 ± 0.0817 |
| Pyrocatechol | 0.2660 ± 0.0017 | 0.2663 ± 0.0061 | 0.2652 ± 0.0034 | 0.2684 ± 0.0015 | 0.2654 ± 0.0071 | 0.2648 ± 0.0097 | 0.2658 ± 0.0056 | 0.2693 ± 0.0012 | 0.2689 ± 0.0089 | 0.2647 ± 0.0042 | 0.2665 ± 0.0017 |
| Methyl gallate | 0.7960 ± 0.0021 | 0.7943 ± 0.0018 | 0.7967 ± 0.0083 | 0.7952 ± 0.0017 | 0.7977 ± 0.0059 | 0.7911 ± 0.0076 | 0.7943 ± 0.0038 | 0.7953 ± 0.0076 | 0.7966 ± 0.0085 | 0.7984 ± 0.0028 | 0.7956 ± 0.0021 |
| Hydroxypaeoniflorin | 1.0150 ± 0.0018 | 1.0144 ± 0.0024 | 1.0149 ± 0.0032 | 1.0145 ± 0.0043 | 1.0152 ± 0.0099 | 1.0192 ± 0.0047 | 1.0176 ± 0.0052 | 1.0141 ± 0.0014 | 1.0151 ± 0.0061 | 1.0134 ± 0.0027 | 1.0153 ± 0.0018 |
| Aesculetin | 4.9691 ± 0.0053 | 4.9672 ± 0.0108 | 4.9687 ± 0.0096 | 4.9677 ± 0.0112 | 4.9690 ± 0.0078 | 4.9854 ± 0.0084 | 4.9694 ± 0.0073 | 4.9698 ± 0.0181 | 4.9695 ± 0.0057 | 4.9696 ± 0.0049 | 4.9705 ± 0.0053 |
| Caffeic acid | 0.5279 ± 0.0008 | 0.5284 ± 0.0006 | 0.5292 ± 0.0012 | 0.5280 ± 0.0016 | 0.5263 ± 0.0034 | 0.5276 ± 0.0016 | 0.5273 ± 0.0024 | 0.5274 ± 0.0015 | 0.5279 ± 0.0013 | 0.5269 ± 0.0026 | 0.5277 ± 0.0008 |
| Albiflorin | 4.6362 ± 0.0110 | 4.6352 ± 0.0145 | 4.6355 ± 0.0176 | 4.6367 ± 0.0128 | 4.6377 ± 0.0216 | 4.6359 ± 0.0156 | 4.6363 ± 0.0097 | 4.6361 ± 0.0119 | 4.6373 ± 0.0065 | 4.6383 ± 0.0078 | 4.6365 ± 0.0010 |
| Procyanidine | 2.3297 ± 0.0032 | 2.3283 ± 0.0106 | 2.3296 ± 0.0145 | 2.3298 ± 0.0098 | 2.3292 ± 0.0112 | 2.3296 ± 0.0078 | 2.3293 ± 0.0046 | 2.3277 ± 0.0051 | 2.3282 ± 0.0101 | 2.3389 ± 0.0023 | 2.3300 ± 0.0032 |
| Paeoniflorin | 8.9277 ± 0.0256 | 8.9213 ± 0.1262 | 8.9267 ± 0.0897 | 8.9246 ± 0.0573 | 8.9279 ± 0.0789 | 8.9264 ± 0.0923 | 8.9332 ± 0.0854 | 8.9421 ± 0.0842 | 8.9278 ± 0.0957 | 8.9281 ± 0.1106 | 8.9286 ± 0.0056 |
| *p*-coumaric acid | 0.3401 ± 0.0029 | 0.3431 ± 0.0046 | 0.3385 ± 0.0032 | 0.3405 ± 0.0019 | 0.3402 ± 0.0015 | 0.3401 ± 0.0078 | 0.3491 ± 0.0091 | 0.3414 ± 0.0083 | 0.3422 ± 0.0076 | 0.3406 ± 0.0095 | 0.3416 ± 0.0029 |
| Benzoic acid | 7.1773 ± 0.0114 | 7.1769 ± 0.0342 | 7.1774 ± 0.0154 | 7.1779 ± 0.0263 | 7.1773 ± 0.0372 | 7.1792 ± 0.0712 | 7.1762 ± 0.0189 | 7.1802 ± 0.0664 | 7.1764 ± 0.0818 | 7.1800 ± 0.0471 | 7.1779 ± 0.0014 |
| Ferulic acid | 0.1419 ± 0.0014 | 0.1413 ± 0.0012 | 0.1423 ± 0.0008 | 0.1436 ± 0.0012 | 0.1433 ± 0.0016 | 0.1401 ± 0.0018 | 0.1453 ± 0.0015 | 0.1418 ± 0.0009 | 0.1422 ± 0.0011 | 0.1434 ± 0.0013 | 0.1425 ± 0.0014 |
| 2,4-dihydroxyacetophenone | 0.2998 ± 0.0030 | 0.2995 ± 0.0023 | 0.2994 ± 0.0041 | 0.2976 ± 0.0023 | 0.2982 ± 0.0019 | 0.2988 ± 0.0024 | 0.2955 ± 0.0035 | 0.2997 ± 0.0029 | 0.2913 ± 0.0021 | 0.2933 ± 0.0027 | 0.2973 ± 0.0030 |
| Gallogen | 4.7710 ± 0.0154 | 4.7684 ± 0.0142 | 4.7754 ± 0.0217 | 4.7582 ± 0.0418 | 4.7723 ± 0.0424 | 4.7717 ± 0.0138 | 4.7710 ± 0.0287 | 4.7708 ± 0.0216 | 4.7754 ± 0.0487 | 4.7777 ± 0.0491 | 4.7712 ± 0.0054 |
| 1,2,3,6-tetra-O-galloyl-β-D-glucose | 1.2149 ± 0.0047 | 1.2126 ± 0.0039 | 1.2159 ± 0.0096 | 1.2283 ± 0.0086 | 1.2143 ± 0.0071 | 1.2167 ± 0.0065 | 1.2143 ± 0.0078 | 1.2185 ± 0.0048 | 1.2219 ± 0.0035 | 1.2146 ± 0.0053 | 1.2172 ± 0.0047 |
| 1,2,3,4,6-O-pentagalloyl glucose | 20.9119 ± 0.1156 | 20.9107 ± 0.1254 | 20.9129 ± 0.1089 | 20.9122 ± 0.1076 | 20.9385 ± 0.1247 | 20.9517 ± 0.1328 | 20.9216 ± 0.1423 | 20.9319 ± 0.1157 | 20.9119 ± 0.1125 | 20.9005 ± 0.1327 | 20.9204 ± 0.0156 |
| Apigenin 7-O-neohesperidoside | 0.4480 ± 0.0025 | 0.4481 ± 0.0013 | 0.4467 ± 0.0008 | 0.4481 ± 0.0011 | 0.4491 ± 0.0023 | 0.4438 ± 0.0019 | 0.4471 ± 0.0054 | 0.4470 ± 0.0023 | 0.4483 ± 0.0039 | 0.4410 ± 0.0033 | 0.4467 ± 0.0025 |
| Apigenin-7-O-glucoside | 0.6035 ± 0.0012 | 0.6041 ± 0.0009 | 0.6037 ± 0.0098 | 0.6035 ± 0.0054 | 0.6037 ± 0.0032 | 0.6049 ± 0.0075 | 0.6045 ± 0.0086 | 0.6056 ± 0.0035 | 0.6072 ± 0.0042 | 0.6050 ± 0.0054 | 0.6046 ± 0.0012 |
| Mudanpioside C | 0.3333 ± 0.0011 | 0.3349 ± 0.0014 | 0.3322 ± 0.0019 | 0.3348 ± 0.0022 | 0.3337 ± 0.038 | 0.3358 ± 0.0031 | 0.3342 ± 0.0037 | 0.3352 ± 0.0029 | 0.3355 ± 0.0026 | 0.3339 ± 0.0024 | 0.3343 ± 0.0011 |
| Paeonol | 0.1288 ± 0.0009 | 0.1291 ± 0.0004 | 0.1284 ± 0.0002 | 0.1290 ± 0.0015 | 0.1291 ± 0.0001 | 0.1278 ± 0.0007 | 0.1265 ± 0.0016 | 0.1299 ± 0.0003 | 0.1289 ± 0.0023 | 0.1289 ± 0.0024 | 0.1286 ± 0.0009 |

**Supplementary Table S3. Content of the 21 components of EMB across 10 batches (n = 3, mean ± SD, mg.g^−1^).**

| Component | S1 | S2 | S3 | S4 | S5 | S6 | S7 | S8 | S9 | S10 | Mean |
| --- | --- | --- | --- | --- | --- | --- | --- | --- | --- | --- | --- |
| Vitamin B6 | 1.4538 ± 0.0218 | 1.4691 ± 0.0106 | 1.4554 ± 0.0138 | 1.4472 ± 0.0212 | 1.4349 ± 0.0245 | 1.4521 ± 0.0271 | 1.4601 ± 0.0109 | 1.4698 ± 0.0278 | 1.4732 ± 0.0283 | 1.4724 ± 0.0112 | 1.4588 ± 0.0125 |
| Gallic acid | 54.0711 ± 0.2313 | 54.5722 ± 0.3354 | 54.3156 ± 0.2984 | 54.4183 ± 0.2832 | 54.2065 ± 0.2389 | 54.5546 ± 0.2725 | 54.8212 ± 0.3429 | 54.9468 ± 0.3123 | 54.0045 ± 0.2812 | 54.0075 ± 0.2213 | 54.3918 ± 0.3321 |
| Pyrocatechol | 0.1516 ± 0.0051 | 0.1512 ± 0.0003 | 0.1519 ± 0.0076 | 0.1512 ± 0.0035 | 0.1515 ± 0.0081 | 0.1516 ± 0.0001 | 0.1546 ± 0.0018 | 0.1567 ± 0.0024 | 0.1542 ± 0.0005 | 0.1538 ± 0.0013 | 0.1518 ± 0.0005 |
| Methyl gallate | 0.5813 ± 0.0056 | 0.5895 ± 0.0075 | 0.5854 ± 0.0087 | 0.5813 ± 0.0065 | 0.5863 ± 0.0084 | 0.5864 ± 0.0023 | 0.5875 ± 0.0045 | 0.5882 ± 0.0073 | 0.5898 ± 0.0078 | 0.5894 ± 0.0058 | 0.5865 ± 0.0031 |
| Hydroxypaeoniflorin | 1.8638 ± 0.0102 | 1.8549 ± 0.0215 | 1.8676 ± 0.0186 | 1.8625 ± 0.0127 | 1.8668 ± 0.0183 | 1.8797 ± 0.0214 | 1.8712 ± 0.0211 | 1.8549 ± 0.0225 | 1.8833 ± 0.0172 | 1.8365 ± 0.0251 | 1.8641 ± 0.0134 |
| Aesculetin | 5.1455 ± 0.0526 | 5.1231 ± 0.0476 | 5.2175 ± 0.0485 | 5.2216 ± 0.0538 | 5.1893 ± 0.0613 | 5.2341 ± 0.0378 | 5.1467 ± 0.0415 | 5.1487 ± 0.0254 | 5.1443 ± 0.0277 | 5.1848 ± 0.0316 | 5.1756 ± 0.0391 |
| Caffeic acid | 0.2428 ± 0.0026 | 0.2443 ± 0.0015 | 0.2414 ± 0.0023 | 0.2435 ± 0.0027 | 0.2476 ± 0.0031 | 0.2416 ± 0.0025 | 0.2423 ± 0.0013 | 0.2447 ± 0.0017 | 0.2435 ± 0.0028 | 0.2429 ± 0.0031 | 0.2435 ± 0.0018 |
| Albiflorin | 3.2695 ± 0.0245 | 3.264 ± 0.0238 | 3.2656 ± 0.0415 | 3.2678 ± 0.0423 | 3.2712 ± 0.0391 | 3.2768 ± 0.0184 | 3.2546 ± 0.0275 | 3.2438 ± 0.0388 | 3.2126 ± 0.0456 | 3.2763 ± 0.0379 | 3.2603 ± 0.0195 |
| Procyanidine | 5.0750 ± 0.0624 | 5.0734 ± 0.0512 | 5.0798 ± 0.0345 | 5.1056 ± 0.0477 | 5.2073 ± 0.0529 | 5.1066 ± 0.0387 | 5.0734 ± 0.0581 | 5.0983 ± 0.0362 | 5.1137 ± 0.0237 | 5.1254 ± 0.0613 | 5.1059 ± 0.0402 |
| Paeoniflorin | 15.2799 ± 0.1143 | 15.2564 ± 0.1217 | 15.3865 ± 0.0987 | 15.2438 ± 0.1457 | 15.6799 ± 0.1509 | 15.2732 ± 0.1361 | 15.5876 ± 0.1416 | 15.2542 ± 0.1339 | 15.4108 ± 0.1247 | 15.4365 ± 0.1124 | 15.3809 ± 0.1521 |
| *p*-coumaric acid | 0.2131 ± 0.0023 | 0.2165 ± 0.0012 | 0.2142 ± 0.0023 | 0.2159 ± 0.0015 | 0.2144 ± 0.0017 | 0.2141 ± 0.0019 | 0.2132 ± 0.0018 | 0.2152 ± 0.0027 | 0.2157 ± 0.0021 | 0.2148 ± 0.0019 | 0.2147 ± 0.0011 |
| Benzoic acid | 7.9380 ± 0.1025 | 7.9323 ± 0.0876 | 7.9765 ± 0.1127 | 7.9651 ± 0.1243 | 7.9380 ± 0.0985 | 7.9998 ± 0.1219 | 7.9845 ± 0.0734 | 7.8832 ± 0.0598 | 7.7685 ± 0.0473 | 7.9347 ± 0.1182 | 7.9321 ± 0.0664 |
| Ferulic acid | 0.1012 ± 0.0016 | 0.10230 ± 0.0013 | 0.1009 ± 0.0015 | 0.1014 ± 0.0.0006 | 0.1018 ± 0.0011 | 0.1025 ± 0.0017 | 0.1021 ± 0.0019 | 0.1017 ± 0.0009 | 0.1013 ± 0.0004 | 0.1016 ± 0.0014 | 0.1017 ± 0.0005 |
| 2,4-dihydroxyacetophenone | 0.1191 ± 0.0015 | 0.1194 ± 0.0023 | 0.1193 ± 0.0018 | 0.1187 ± 0.0021 | 0.1182 ± 0.0016 | 0.1188 ± 0.0012 | 0.1179 ± 0.0005 | 0.1177 ± 0.0007 | 0.1185 ± 0.0017 | 0.1189 ± 0.0014 | 0.1187 ± 0.0006 |
| Gallogen | 19.3970 ± 0.2235 | 19.5394 ± 0.2256 | 19.4235 ± 0.2176 | 19.1398 ± 0.1884 | 19.6613 ± 0.2025 | 19.1877 ± 0.1927 | 19.9143 ± 0.1924 | 19.3986 ± 0.2116 | 19.3982 ± 0.2019 | 19.3884 ± 0.2347 | 19.4448 ± 0.2225 |
| 1,2,3,6-tetra-O-galloyl-β-D-glucose | 1.0377 ± 0.0081 | 1.0216 ± 0.0114 | 1.0332 ± 0.0024 | 1.0378 ± 0.0087 | 1.0385 ± 0.0095 | 1.0345 ± 0.0068 | 1.0428 ± 0.0027 | 1.0414 ± 0.0075 | 1.0388 ± 0.0082 | 1.0392 ± 0.0065 | 1.0366 ± 0.0060 |
| 1,2,3,4,6-O-pentagalloyl glucose | 7.3362 ± 0.0876 | 7.3546 ± 0.1023 | 7.3768 ± 0.0915 | 7.4713 ± 0.0865 | 7.5438 ± 0.0928 | 7.2163 ± 0.0744 | 7.2357 ± 0.0738 | 7.3389 ± 0.1105 | 7.3343 ± 0.0997 | 7.3355 ± 0.0863 | 7.3543 ± 0.0969 |
| Apigenin 7-O-neohesperidoside | ND | ND | ND | ND | ND | ND | ND | ND | ND | ND | ND |
| Apigenin-7-O-glucoside | 2.2858 ± 0.0127 | 2.2756 ± 0.0217 | 2.2623 ± 0.0234 | 2.2814 ± 0.0304 | 2.2872 ± 0.0187 | 2.2915 ± 0.0149 | 2.2944 ± 0.0216 | 2.2785 ± 0.0183 | 2.2655 ± 0.0152 | 2.2427 ± 0.0188 | 2.2765 ± 0.0158 |
| Mudanpioside C | 0.5361 ± 0.0032 | 0.5345 ± 0.0056 | 0.5367 ± 0.0072 | 0.5355 ± 0.0065 | 0.5368 ± 0.0039 | 0.5332 ± 0.0072 | 0.5378 ± 0.0013 | 0.5397 ± 0.0024 | 0.5325 ± 0.0081 | 0.5314 ± 0.0042 | 0.5354 ± 0.0025 |
| Paeonol | 0.1727 ± 0.0012 | 0.1724 ± 0.0008 | 0.1703 ± 0.0011 | 0.1734 ± 0.0023 | 0.1721 ± 0.0017 | 0.1716 ± 0.0018 | 0.1711 ± 0.0007 | 0.1728 ± 0.0022 | 0.1715 ± 0.0019 | 0.1713 ± 0.0018 | 0.1719 ± 0.0009 |

* not detected.
